# Prevalence and Clinical Characteristics of Low Back Pain among Operating Room Personnel: A Cross-Sectional Study in South of Iran

**DOI:** 10.3389/fsurg.2022.841339

**Published:** 2022-05-27

**Authors:** Reza Fayzi, Ashkan Karimi, Armin Fereidouni, Armin Salavatian, Behzad Imani, Reza Tavakkol

**Affiliations:** ^1^ Department of Operating Room, School of Paramedical, Kermanshah University of Medical Science, Kermanshah, Iran; ^2^ Department of Operating Room, School of Paramedician, Hamadan University of Medical Science, Hamadan, Iran; ^3^ Department of Operating Room Technology, School of Nursing and Midwifery, Shiraz University of Medical Sciences, Shiraz, Iran; ^4^ Students Research Committee, School of Paramedical, Kermanshah University of Medical Sciences, Kermanshah, Iran; ^5^ Department of Operating Room, School of Paramedician, Hamadan University of Medical Science, Hamadan, Iran; ^6^ Nursing and Midwifery Care Research Center, Mashhad University of Medical Sciences, Mashhad, Iran

**Keywords:** musculoskeletal disorders, low back pain, operating room, personnel, surgery, perioperative nurse

## Abstract

**Background:**

Low back pain is one of the most common musculoskeletal disorders and the most common cause of activity restriction in people younger than 45 years. Nurses have a high incidence and prevalence of low back pain in terms of physical and emotional factors among healthcare workers. This study aimed to evaluate the prevalence and clinical characteristics of low back pain.

**Methods:**

This cross-sectional study was performed on 385 operating room personnel of the hospitals affiliated with Shiraz (the largest city in southern Iran) University of Medical Sciences. The data were collected via a research-made developed questionnaire. The questionnaire consisted of two parts, including the demographic information and prevalence and dimensions of low back pain.

**Results:**

The results showed that the prevalence of low back pain was 74% among operating room personnel. There was a significant relationship among low back pain, education level, and marital status (*p* < 0.05).

**Conclusion:**

Hospital managers should reduce the prevalence of this disorder among operating room staff by teaching preventative patient handling techniques via training courses.

## Introduction

Low back pain (LBP) is one of the most common types of work-related musculoskeletal disorders (MSDs) (1, 2). LBP is an important public health subject with high prevalence and a considerable negative physical, psychological, social, and economic impact (3–5). In general, this disorder due to the duration of pain includes three categories: acute (less than 6 weeks), subacute (from 6 to 12 weeks), and chronic (more than 12 weeks) (6, 7).

The peak of LBP is in the third decade of life, and its incidence increases to 60–65 years and then gradually decreases (8, 9). Nearly more than half of the general population will experience LBP care at some point in their lives (10). The prevalence of LBP among the general population is estimated to be from 15% to 45% (11). Thirty-seven percent of LBP is related to occupations in which professionals are exposed to prolonged periods of standing, such as healthcare workers (3). LBP can be caused by various components of the spine, abdominal structures, or pelvic organ (12). Among healthcare workers, nurses have a high incidence of LBP (13) due to physical and emotional factors (14). Considering the prolonged standing, unpleasant physical conditions, and movement limitations during surgery (15), the prevalence of LBP was reported in 70.6% of operating room personnel (16) and most other parts of the hospital (17).

The purpose of this research was to determine the frequency of LBP and clinical features among operating room staff at Shiraz University of Medical Sciences. The findings of this research will be presented to hospital administrators and operating room directors, who will be able to design suitable initiatives to prevent, control and reduce the recurrence of low back pain in operating room staff.

## Method

### Study Design

This is a cross-sectional study performed in 2019 on operating room personnel working in hospitals affiliated with Shiraz (the largest city in southern Iran) University of Medical Sciences, including Faghihi, Namazi, Khalili, Rajaii, Chamran, and Amiralmomenin hospitals.

### Population and Sample Size

A total of 385 operating room personnel were sampled, similar to prior research by Choobineh et al. (18), the sample size was determined with average prevalence of low back pain 50%, confidence estimates of 95%, and 0.05 accuracy.

### Data Collection

The total number of operating room personnel in each hospital was first determined, and then, the personnel were recruited and selected for participation based on stratified random sampling. To this end, we referred to investigators who administered surveys in hospitals during different shifts after obtaining permission from the Ethics Committee, and the sampling was carried out based on the inclusion criteria mentioned in different shifts after obtaining informed and written consent from the personnel. The questionnaires were distributed among the personnel and collected at the end of the office time on the same day.

### Inclusion and Exclusion Criteria

Participants’ inclusion criteria included a willingness to participate in the study and 2 years of work experience. Exclusion criteria were osteoporosis, acquired spinal diseases, and corticosteroid consumption.

### Measuring Instruments

Research-made developed questionnaire was used to evaluate and gather data on the prevalence and presence of LBP and its related dimensions. The validity and reliability of the questionnaire had already been examined. During the psychometric phase of instrument development, qualitative and quantitative methods were used to determine the face validity and content and construct validity evaluation. The face validity of qualitative data was checked by interviews with 20 professors and experts in this field, resulting in a modification of three items and the addition of two items. During the quantitative face validity phase, which assessed the importance of each item by using the item impact formula, all tool items received the least impact score. This was done by filling out the initial questionnaire by 20 operating room personnel. The content validity of the qualitative section was determined by the experts’ correction; three items were corrected, and one item was merged with the other items. Quantitative evaluation of the content validity was done using two indices of the content validity ratio and content validity index, by 14 and 10 professors and experts in this field, respectively. In this phase, two items failed to achieve the required score and were eliminated. The construct validity assessment was completed via the convergent and divergent validity, respectively, using the chronic pain questionnaire and General Health Questionnaire distributed among 30 operating room personnel. The correlation of the mentioned instruments was investigated with the research instrument. The relationship between the research instrument with the chronic pain questionnaire as a convergent tool and the General Health Questionnaire as a divergent instrument was 0.70 and −0.37, respectively, which was statistically significant (*p* < 0.05). The reliability and internal consistency of the instrument, determined by test–retest and Cronbach’s alpha coefficient, were found to be 0.77 and 0.60, respectively. The questionnaire consisted of two sections: “Demographic information and prevalence and dimensions of LBP.” This questionnaire assessed the prevalence and dimensions of LBP using 11 multiple-choice questions (18).

### Data Analyses

After collecting the questionnaires, the data were entered into SPSS version 19 and then analyzed. Descriptive statistics calculated demographic data of the population, frequency distribution, mean, and standard deviation.

### Ethical Considerations

The Institutional Review Board of Shiraz University approved this medical science study (ethical code: IR.SUMS.REC.1398.263) available link:https://ethics.research.ac.ir/EthicsProposalView.php?id = 57702.

All participants agreed to participate in the study and signed informed consent. The participants were assured that their information would remain confidential.

## Results

A total of 385 operating room personnel working in six hospitals affiliated with Shiraz University of Medical Sciences were studied. Table 1 shows the frequency distribution of the demographic characteristics of the population under study. Among 385 participants, 240 (62.3%) were women. The mean and standard deviation of the age was 33.25 ± 7 7.96. Besides, the majorities of participants were undergraduates (85.7%) and married (57.4%).

**Table 1:** Demographic characteristics of the operating room personnel.

	Variable	Frequency (%)
Gender	Male	240 (62.3)
	Female	145 (37.7)
Age	20–29 years	155 (40.3)
	30–39 years	150 (39)
	40–49 years	58 (15.1)
	Above 50 years	22 (5.7)
Marital status	Married	221 (57.4)
	Single	162 (42.6)
Education level	Bachelor degree	330 (85.7)
	Associate degree	45 (11.7)
	Master degree	10 (2.6)

The prevalence of LBP was reported at 75%. Therefore, it was found that the prevalence of LBP in hospitals varied, and the highest prevalence (84.2%) was in the hospital of burn and plastic (Table 2). Also, the highest prevalence of LBP was in married (61.05%) people.

**Table 2:** Prevalence of LBP in the studied hospitals.

Hospital name	Number of personnel	Prevalence of low back pain, frequency (%)
Faghihi	83	62 (74.6)
Namazee	94	62 (65.9)
Khalili	49	37 (75.5)
Rajaey	85	68 (80)
Chamran	55	40 (72.7)
Amiralmomenin	19	16 (84.2)
Total	385	285 (74)

Table 3 shows the clinical characteristics of low back pain in the participants of this study. Most participants had a normal body mass index (BMI) (73.2%), work experience of less than 10 years (60.3%), rotating shifts (71.7%), and standing physical condition (54%). In addition, in 67.71% of participants, low back pain occurred less than 10 years after the start of work, and 81.40% of total low back pain was acute low back pain in nature. Sixty-seven percent of the patients reported pain only in the lumbar region, and according to the type of pain, intermittent pain was the most frequent, with 32.28%. Another criterion evaluated in this study was the severity of pain in the patients. Furthermore, transient mild pain that resolves spontaneously without using analgesics or rest was the highest, with 60.35%. The relationship between participant demographic information and clinical characteristics of low back pain showed that only sex (*p*-value = 0.023) and marital status (*p*-value = 0.017) had a significant relationship with pain area (Table 4).

**Table 3:** Clinical characterization of low back pain among operating room personnel.

	Variable	Frequency (%)
BMI	BMI is less than 18.5 (underweight)	20 (5.2%)
	BMI is 18.5 to 24.9 (normal weight)	282 (73.2%)
	BMI is 25 to 29.9 (overweight)	77 (20%)
	BMI is 30 or more (obese)	6 (1.6%)
Work experience	Less than 10 years	232 (60.3%)
	10–20 years	109 (28.3%)
	More than 20 years	44 (11.4%)
Shifts	Morning	60 (15.6%)
	Evening	33 (8.6%)
	Night	16 (4.2%)
	Rotating Shift	276 (71. 7%)
Physical condition	Standing	208 (54%)
	Sitting	37 (9.6%)
	Moving	21 (5.5%)
	Combination of different physical conditions	119 (30.%9)
Duration of pain	Acute (less than 6 weeks)	232 (81.40%)
	Subacute (between 6 and 12 weeks)	34 (11.92%)
	Chronic (more than 12 weeks)	1 (0.35%)
	No improvement	18 (6.31%)
Beginning of low back pain	Less than 5 years	193 (67.71%)
	5–9 years	69 (24.21%)
	10–15 years	21 (7.36%)
	More than 15 years	2 (0.70%)
Pain area	Regional low back pain (only in the waist area)	191 (67.01%)
	Low back pain with spread to the legs	94 (32.98)
Type of pain	Sharp pain	23 (8.07%)
	Burning pain	36 (12.63%)
	Stingray and mormor	76 (26.66%)
	Numbness	13 (4.56%)
	Intermittent pain	92 (32.28%)
	Constant pain	26 (9.12%)
	Constant exacerbating pain	7 (2.45%)
	Other cases	12 (4.21%)
Severity of pain	Transient mild pain that resolves spontaneously without the use of analgesics or rest	172 (60.35%)
	Permanent mild pain in such a way that it does not stop you from doing the hard work	62 (21.75%)
	Moderate pain in such a way as to prevent heavy physical activity or heavy work	44 (15.43%)
	Severe pain that prevents daily activities	7 (2.45%)

**Table 4:** The relationship between clinical characteristics of low back pain and demographic characteristics of the operating room personnel.

Variable		Frequency (%)	The significance level
Gender	Male	240 (62.3)	0.023
	Female	145 (37.7)	
Marital status	Married	221 (57.4)	0.017
	Single	162 (42.6)	

## Discussion

The present study was performed to evaluate the prevalence of LBP and its clinical characterization in operating room personnel of six hospitals affiliated with Shiraz University of Medical Sciences. This study’s results showed a high prevalence of low back pain (75%) in operating room personnel. LBP is one of the most common work-related MSDs (19), which has many detrimental consequences for the healthcare system and its personnel (20).

LBP had a high prevalence among participants. In a study conducted by Choobineh et al. to investigate the prevalence of MSDs in the Shiraz operating room nurses, it was found that more than half of the participants had LBP during the past year, which was the most prevalent among other MSDs (18). High prevalence can be attributed to working in a stressful operating room environment and specific activities. In another study, Trinkoff et al. reported LBP as the most common musculoskeletal disorder in nurses at the University of Maryland, USA (21). The prevalence of LBP was higher in women, indicating the impact of the female sex on the development of low back pain (8).

The other additional results of this study revealed differentiated pain duration. Consistent with this finding, 167 patients had acute LBP and a few suffered from chronic LBP. In the study by La Torre et al., LBP was identified as the most important musculoskeletal disorder among operating room nurses in Rome, and acute LBP was the most prevalent (23). The high prevalence of acute LBP in these studies may be in terms of the heavy and risky transient activity during surgical operations, such as stretching and specific physical postures in operating room personnel. Besides, in a study conducted by Barkhordari et al. to evaluate the prevalence of LBP in nurses of hospitals in Yazd, the duration of pain in LBP patients was in two-thirds cases less than two weeks and in others at least two weeks during the past 12 months (24).

Other results of this study indicated the absence of work in one-third of the affected people. Moreover, about two-thirds of patients did not see a doctor. Among those who were referred to a physician, most people underwent diagnostic or therapeutic measures. Drug therapy and X-rays were the most prevalent diagnostic modality among these measures. In a similar study, Bejia et al. reported the prevalence of LBP in more than half of Monastir Tunisian hospital staff. About one-quarter of patients in this study suffered from chronic low back pain. Thus, it was found that most patients needed medical care, and about half of them needed radiologic diagnoses. Hence, in a significant number of participants, LBP led to absence from work (25). Regarding the high rate of chronic LBP in the study of Bejia compared to the present study, there may be an increased need for patients with medical care and radiological diagnoses, as well as an increased absence of work related to this type of LBP.

Another notable result of this study was determining the severity of LBP and its impact on participants’ daily life activities. The results showed that more than half of the patients had transient and permanent mild pain, about two-fifths of them had moderate pain that prevented heavy physical activity or heavy work, and only a few suffered severe pain that prevented them from doing their daily activities. In a study conducted by Awosan et al. to investigate the prevalence of LBP among healthcare workers in Sokoto, Nigeria, most participants experienced LBP after work. Among the patients, about half had mild pain, a significant number had moderate pain, and a small number had severe pain (26). By comparing the results of the Awosan study and the present study, it was found that most patients had mild pain. High-risk activities are probably the reason for increased pain over time, while rest leads to decreased pain intensity and less progression towards chronic forms.

### Limitations

The limitations of this study include no evaluation of students and operating room personnel of private hospitals. Therefore, evaluating other risk factors such as mental status and occupational stress can provide better results among operating room personnel. Besides, it is recommended that other studies should evaluate students and private hospitals. The self-reported surveys were completed by the study subjects and are subject to recall and response bias. Subjects may not have recalled events or characteristics accurately or may have been reluctant to report the truth. This is because of the fact that some people believe that one with LBP lacks a normal life, leading to their decreased self-confidence. This can be due to factors such as old age, high BMI, low education level, and so on, which are related to LBP.

## Conclusions

The present study showed a high prevalence of low back pain among the operating room personnel. By examining the different factors and characteristics of participants, there was a significant relationship among LBP, education level, and marital status. Therefore, more attention is needed to these cases, and the increase in awareness among operating room staff via taking classes and courses has partly reduced the prevalence of the disorder.

## Data Availability

The raw data supporting the conclusions of this article will be made available by the authors without undue reservation.
